# Interior-architectured ZnO nanostructure for enhanced electrical conductivity via stepwise fabrication process

**DOI:** 10.1186/1556-276X-9-428

**Published:** 2014-08-24

**Authors:** Eugene Chong, Sarah Kim, Jun-Hyuk Choi, Dae-Geun Choi, Joo-Yun Jung, Jun-Ho Jeong, Eung-sug Lee, Jaewhan Lee, Inkyu Park, Jihye Lee

**Affiliations:** 1 Department of Nano Manufacturing Technology, Korea Institute of Machinery and Materials, Daejeon 305-343, South Korea; 2 Nano-mechatronics Department, University of Science and Technology (UST), Daejeon 305-333, South Korea; 3 Department of Mechanical Engineering and KI for the NanoCentury, Korea Advanced Institute of Science and Technology (KAIST), Daejeon 305-701, South Korea; 4 Current address: Agency for Defense Development (ADD), Daejeon, South Korea

**Keywords:** Electrical conductivity, Interior-architecturing, ZnO nanostructure, Nanoimprint Lithography(NIL), Zinc oxide (ZnO), Hydrothermal growth

## Abstract

Fabrication of ZnO nanostructure via direct patterning based on sol-gel process has advantages of low-cost, vacuum-free, and rapid process and producibility on flexible or non-uniform substrates. Recently, it has been applied in light-emitting devices and advanced nanopatterning. However, application as an electrically conducting layer processed at low temperature has been limited by its high resistivity due to interior structure. In this paper, we report interior-architecturing of sol-gel-based ZnO nanostructure for the enhanced electrical conductivity. Stepwise fabrication process combining the nanoimprint lithography (NIL) process with an additional growth process was newly applied. Changes in morphology, interior structure, and electrical characteristics of the fabricated ZnO nanolines were analyzed. It was shown that filling structural voids in ZnO nanolines with nanocrystalline ZnO contributed to reducing electrical resistivity. Both rigid and flexible substrates were adopted for the device implementation, and the robustness of ZnO nanostructure on flexible substrate was verified. Interior-architecturing of ZnO nanostructure lends itself well to the tunability of morphological, electrical, and optical characteristics of nanopatterned inorganic materials with the large-area, low-cost, and low-temperature producibility.

## Background

Zinc oxide (ZnO) has been widely pursued due to its electronic and optoelectronic characteristics arising from a direct wide bandgap (Eg ~ 3.37 eV) and an isoelectronic point with a large excitation binding energy (60 meV) at room temperature [1]–[7]. These are sought-after features in a number of electric devices, such as sensor/detectors, light-emitting diodes, solar cells, field-effect transistors (FETs), and nanogenerators [1]–[4]. Recently, much research has been carried out in the field of nanostructured ZnO, investigating one hole-one nanorods, micro/nanodots, patterned seed layers, and nanoparticles. In-depth research results have confirmed that nanostructured ZnO is a promising material in the fields of photonics and electronics.

Increasing the ease of fabricating ZnO nanostructure arrays on almost any substrate would enable large-scale production for a wide range of applications. However, the current commonly applied methods for ZnO deposition, such as chemical vapor deposition (CVD) [8,9], pulsed laser deposition (PLD) [10], metal-organic chemical vapor deposition (MOCVD) [11], and atomic layer deposition (ALD) [12]–[14], require high-cost equipment and high-vacuum conditions, and/or are subject to substrate limitations due to high process temperatures. For the latter reason, hydrothermal synthesis of ZnO has been welcomed due to its low process temperature. However, the resulting structures are generally short and are difficult to integrate to form large arrays. As for fabricating micro/nanosized arrays or nanopatterns, the main methods utilized are electron-beam lithography (EBL) [15,16], photo- lithography (PL) [17,18], and laser interference lithography (LIL) [19]. These processes have their share of limitations too: some are costly and require a controlled environment, while others are difficult to perform on flexible or large substrates.

For these reasons, direct nanopatterning of metal oxides using the sol-gel process such as nanoimprint lithography (NIL) has garnered much attention as a simple, low-cost, and rapid technique potentially suited to large-area fabrication and producibility on flexible or non-uniform substrates. Directly nanoimprinted ZnO layers have been applied in light-emitting devices and advanced nanostructuring [20]–[22]. However, its application as an electrically conducting medium has been limited by its low conductivity, which has been attributed to organic residues and a low degree of crystallinity after low-temperature calcination, or increased porosity after high-temperature calcination. Improving the electrical conductivity of directly nanoimprinted ZnO nanostructures at low process temperatures would open opportunities for their application in low-temperature electronic devices, including flexible devices and allow for large-area mass fabrication.

**Figure 1:**
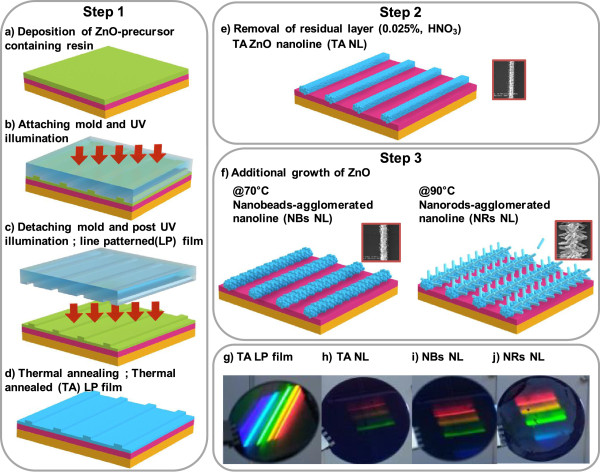
**Fabrication procedure for ordered arrays of ZnO nanolines with improved electrical conductivity. (a)** nanopatterning of ZnO film (step 1), **(b)** wet etching (step 2), and **(c)** additional growth of ZnO nanocrystals (step 3).

Here, we report the interior-architecturing of sol-gel-based ZnO nanostructure for enhanced electrical conductivity via stepwise fabrication process. Stepwise combined method of NIL process with wet etching and hydrothermal growth was applied for the interior-architecturing. Interior low crystalline regions and organic residuals resulting from low-temperature calcinations were removed, and the resultant structural voids filled up with newly growing ZnO nanocrystalline. Figure 1 shows the stepwise fabrication procedure, which is composed of three steps: nanopatterning ZnO film (step 1), wet etching for removal of the residual layer (step 2), and the additional growth of ZnO (step 3). Thermally annealed line-patterned ZnO film was fabricated using direct ultraviolet-NIL (UV-NIL) and thermal annealing (Figure 1a,b,c,d). During wet etching, the residual layer was removed and ordered arrays of individually separated ZnO nanolines were formed (Figure 1e). For the additional growth of ZnO nanograins, hydrothermal synthesis at two different temperatures was applied (Figure 1f). Photos of the resulting substrates with thermally annealed line-patterned ZnO film and ordered arrays of ZnO nanolines are shown in Figure 1g,h,i,j. Their morphologies, crystal structures, and electrical and photoresponsive characteristics were measured and the relationships analyzed. These ordered arrays of ultralong ZnO nanolines were fabricated on both a rigid SiO_2_/Si substrate as well as a flexible polyimide (PI) substrate. The arrays of nanolines were easily integrated as two-electrode nanodevices and performed successfully as UV-sensing devices. Furthermore, fabricated on PI, films were confirmed using a mechanical bending test.

## Methods

### Chemicals: ZnO precursor-containing resin for nanoimprint

ZnO precursor resin was prepared by dissolving 0.5 mol zinc acetate dihydrate (Zn(CH_3_COO)22H_2_O, Aldrich, Wyoming, IL, USA, 99.5%), 2-nitrobenzaldehyde (Aldrich UV-linker), and the molar equivalent of monoethanolamine (MEA, (NH_2_CH_2_CH_2_OH, Aldrich, 99.5%) in 2-methoxyethanol (2ME (CH_3_OCH_2_CH_2_OH, Aldrich, 99.5%). The resulting solution was stirred at 25°C for 3 h and 75°C for 24 h to yield a homogeneous and stable colloid solution.

### Solution for additional growth of ZnO

The solution for hydrothermal synthesis of ZnO nanocrystals was prepared as Zn(NO_3_)_2_ ∙ 6H_2_O (25 mM)-zinc nitrate hexahydrate (Zn(NO_3_)_2_ ∙ 6H_2_O, Aldrich, 98%) in deionized water with HMTA (25 mM)-hexamethylenetetramine (C_6_H_12_N_4_, Aldrich, 99.5%) and PEI (0.834 mM)-polyethylenimine (PEI, Aldrich, molecular weight 1,300 g mol - 1LS).

### Process

#### ***Step 1. Nanopatterning of ZnO film***

Nanopatterning of ZnO was conducted via UV-NIL as follows. First, ZnO precursor-resin was spin-coated onto substrates at 3,500 rpm for 1 min to yield a thickness of 200 nm, then prebaked at 80°C on a hot plate. Si wafer with a 300-nm-thick SiO_2_ layer and polyimide (PI) film were used as the substrates. Next, a nanopatterned polyurethane acrylate (PUA) mold with a regular line pattern array with 200-nm line width and 1-μm period was prepared with the similar method to that previously reported [20]. The mold-attached substrate was then illuminated with 365-nm wavelength light for 3 min to cure the resin, under an applied air pressure of 0.02 MPa. The illumination time of 3 min was set for the resin to be partially cured for the facile detachment of the mold. And then, the PUA mold was detached from the substrate and line patterns were formed on the ZnO resin. After the de-molding, the film was illuminated with 365-nm light for the additional cure of the film and the resultant preservation of the pattern shape during the next thermal annealing. The film was annealed at 350°C for 60 min in a furnace for calcination to take place and cause crystallization. A thermally annealed line-nanopatterned ZnO film resulted, with a residual layer left under the ZnO nanopattern.

#### ***Step 2. Wet etching for removal of residual layer***

To fabricate individually separated ZnO nanoline, the residual layer was removed by wet etching using 0.25% HNO_3_ solution. During removal of the residual layer, some organic components in the ZnO nanopattern originated from ZnO precursor resin were also removed.

#### ***Step 3. Additional growth of ZnO***

Additional ZnO was grown on the ZnO nanostructures by hydrothermal synthesis. The substrate with ZnO nanostructures was kept in the prepared solution for 30 min at 75°C or 90°C in a convention oven. The ZnO nanostructure-deposited surface was positioned so as to be face-down in the solution. After the growth step, the substrate was thoroughly rinsed with deionized water and dried in air.

### Metallization

For the electrical characterization of ZnO nanolines, a single nanoline was connected to Au electrodes via deposition of Pt using a focused ion beam (FIB) system. The Au electrodes were patterned so as to be separated by 2 μm on the SiO_2_/Si substrate prior to deposition of the ZnO resin using lift-off process. For characterization of the ZnO nanostructure arrays, two top electrodes of silver (Ag) were painted through a shadow mask onto the ZnO nanostructure using Ag paste (ELCOAT, P-100, CANS, Japan). The width and length (*W*/*L*) of the gap between the two Ag electrode arrays were 2 and 1 mm, respectively.

### Characterization

The morphology of the ZnO nanolies was investigated using field emission scanning electron microscopy (FE-SEM, FEI co., Hillsboro, OR, USA). X-ray diffraction (XRD, Rigaku, Shibuya-ku, Japan, D/MAX-2500) analysis was used for inspection of the nanostructured ZnO film crystal structure. Diffraction patterns were taken in the 2*θ* range of 10 to 60° using Cu-*Kα* radiation (*λ* = 0.15405 nm), with a scanning rate of 2°/min, step of 0.02°, and incident angle of 3° to the surface. X-ray photoelectron electroscopy measurement was performed in a thermo spectrometer (MultiLab 2000, Thermo Scientific, Waltham, MA, USA; a base pressure of 1 × 10^-9^ Torr) using monochromatized Al-*Kά* radiation. The electrical characteristics of the fabricated ZnO nanostructure and the response to UV illumination were measured with a semiconductor parameter analyzer (Keithley 4200-SCS, Cleveland, OH, USA) at room temperature in a dark room. UV light with 365-nm wavelength was used for illumination. In order to test the mechanical stability of the devices, bending tests were conducted using a single-axis linear stage. The flexible devices were mounted on a carrier substrate and then attached to the linear stage. A bending test with curvature radii from *ρ* = 10 mm to *ρ* = 86 mm was performed over 1,000 cycles while the current was measured at a bias of 5 V using a potentiostat (CHI601D, CH Instruments, Austin, TX, USA).

## Results and discussion

**Figure 2:**
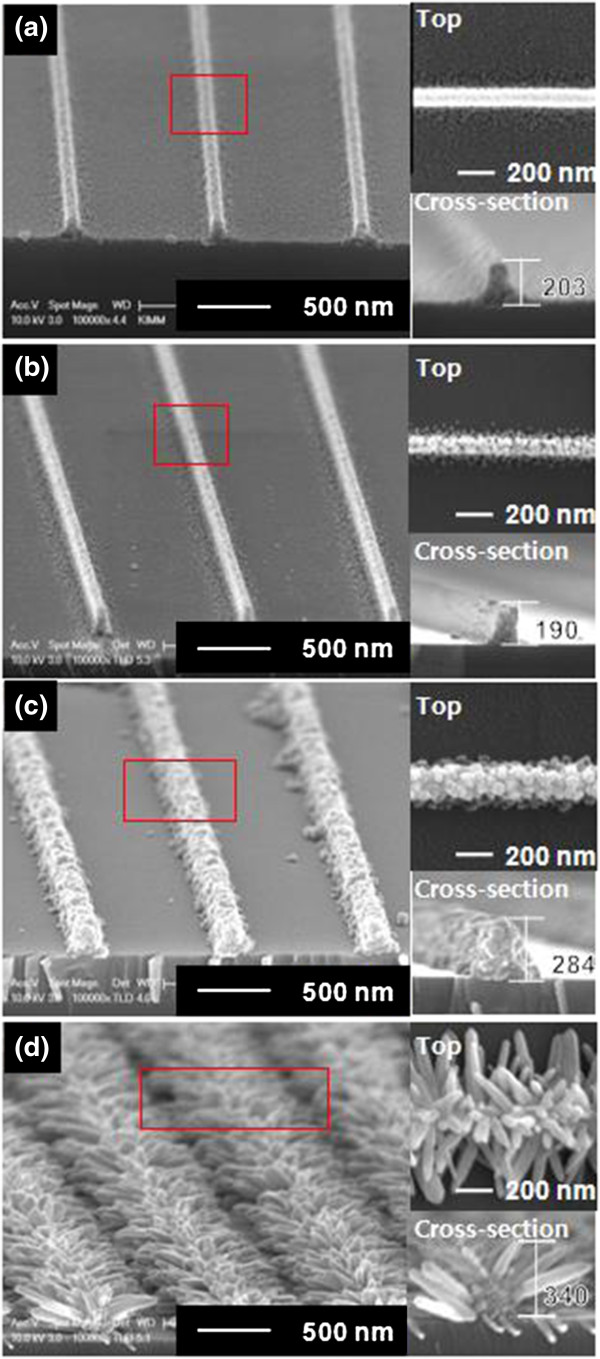
**SEM images of the fabricated ZnO nanostructures.** SEM Images after **(a)** nanopatterning of ZnO film (step 1), **(b)** wet etching (step 2), and **(c, d)** additional growth of ZnO at 75°C (c) and 90°C (d) on SiO_2_/Si substrate.

Figure 2 shows the morphologies of the ZnO nanostructures after each step of the fabrication procedure. Figure 2a shows thermally annealed (TA) line-patterned ZnO film formed after the process step 1. The width and height of the nanolines were approximately 130 and 170 nm, respectively. Line-nanopatterns were coupled to each other by a 30-nm-thick residual layer beneath them. After removal of the residual layer (step 2), individually separated TA nanolines of ZnO were produced (Figure 2b). Magnified views of the top and cross-section show that the surface of the ZnO nanostructure became rougher as organic components and low crystalline regions were simultaneously removed during wet etching. The cross-section shape of TA nanolines shows that wet-etching process was anisotropic while it was supposed to be isotropic generally. It can be ascribed to the difference of porosity between residual layer and line-nanopatterns. Because the porosity of the residual layer was higher than the line-nanopatterns, the residual layer seemed to be removed more quickly than the line-nanopatterns. The results for additional growth of ZnO are shown in Figure 2c,d. The additional growth was performed at two different temperatures: 75°C and 90°C. For 75°C, ZnO was grown from the TA nanolines to form a nanobead (NB)-agglomerated line shape. In terms of crystallographic orientation, ZnO was grown more commonly in the <100 > or <010 > direction, rather than the <002 > direction of the ZnO wurzite structure. The size of the nanograins increased from 15 to 75 nm, and hence, line width also increased from 130 to 180 nm during the additional growth at 75°C. For additional growth at 90°C, ZnO was grown from the TA nanolines to form nanorod(NR)-agglomerated nanolines. Nanorods grew out of line along the TA nanoline like the leaves of a palm tree (Figure 2d). The results indicate that ZnO was grown in the <001 > direction and the overall length of the out-of-line nanorods were around 200 nm.

**Figure 3:**
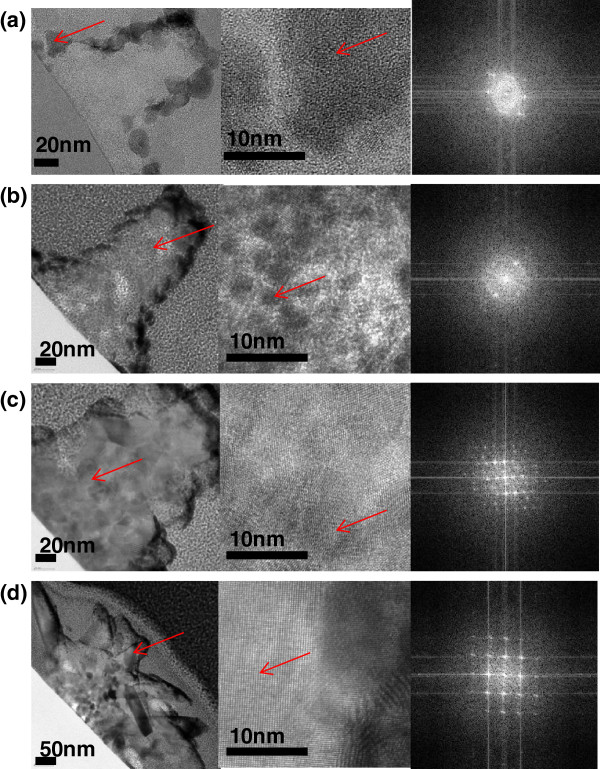
**TEM and FFT images of cross-sections of ZnO nanostructures.** TEM (left and center) and FFT (right) images of cross-sections of ZnO nanostructures after **(a)** nanopatterning of ZnO film, **(b)** wet etching, and the additional growth process at **(c)** 75°C and **(d)** 90°C. The arrows indicate more deeply-investigated areas.

When line-nanopatterned ZnO film was processed to hydrothermal growth at 90°C of step 3 without the wet-etching process of step 2, it appeared that nanorods grew and were buried under the residual layer (Additional file 1: Figure S1). We thus presume that the growth of ZnO was inhibited by the low crystalline region and organic residuals [23].The changes in ZnO nanostructure morphology during the process were investigated using TEM and fast Fourier transform (FFT) images of their cross-sections, as shown in Figure 3. After step 1 of fabrication, the interior nanostructure was composed of both crystalline and amorphous regions (Figure 3a). Figure 3b shows that these amorphous regions were removed during the wet-etching process (step 2), resulting in numerous voids. The additional growth process at 75°C resulted in the growth of nanograins and filling of the voids (Figure 3c). Moreover, the FFT images show that crystallinity increased during the additional growth at 75°C. For additional growth at 90°C, well-ordered single-crystalline ZnO nanorods were grown from the nanograins (Figure 3d). Thus, the low crystalline regions of the ZnO nanostructure from the nanopatterning process were effectively removed during the wet-etching process and were subsequently filled with nanocrystalline ZnO of higher crystallinity.

**Figure 4:**
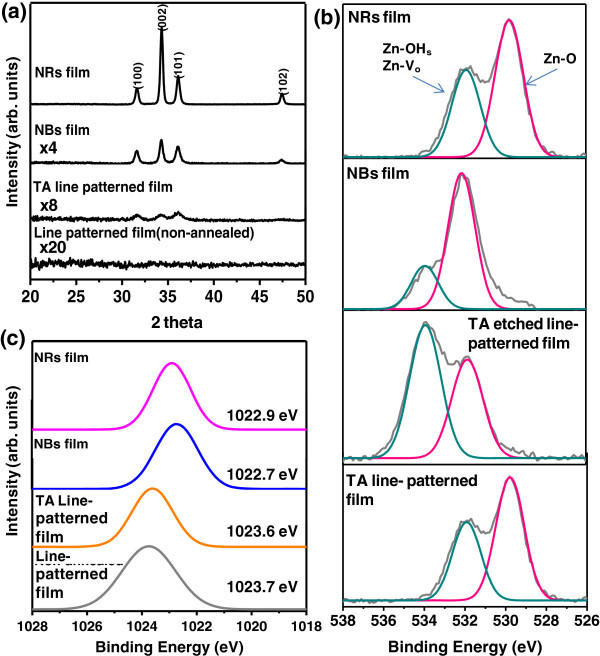
**Structural and elemental analysis of ZnO film at each fabrication step. (a)** XRD patterns, **(b)** XPS analysis of O1s region, and **(c)** Zn2p3 region.

Crystal structures of the fabricated ZnO nanostructures were investigated using XRD and X-ray photoelectron spectroscopy (XPS) analysis. XRD patterns of the nanostructured ZnO films after each fabrication step are plotted in Figure 4a. The crystallographic orientations of the ZnO wurtzite structure are labeled in the XRD patterns [21]. The XRD results indicate that the TA, NB, and NR ZnO films are composed of wurtzite structure crystalline ZnO, with the exception of the line-patterned film before annealing. The degree of crystallinity was higher for the NB and NR films, which can be attributed to the fact that the structural voids were filled with nanocrystalline ZnO. The intensity of the (002) direction for all detected peaks was 0.48 for the NB film, lower than that of the NR film at 0.65. This means that crystal growth in the (002) direction was suppressed at 75°C but dominant at 90°C, which agrees with the morphologies of the fabricated ZnO nanostructure shown in Figure 2c,d.

The chemical binding states measured using XPS are depicted in Figure 4b. The sample surface was pre-cleaned with argon plasma to remove surface contaminants. The presence of C*1s* peaks at 286 eV for all samples is due to contamination by exposure to the atmosphere. The O*1s* peaks showed an asymmetric shape and were deconvoluted into two peaks by Lorentzian-Gaussian spectral fitting [24,25]. Those *O1s* peaks can be assigned to oxide lattices with metal-oxygen bonding (Zn-O), and to oxygen vacancies (Zn-*V*_*O*_), or other hydroxide groups (Zn-OHs) as depicted in Figure 4b. For the TA line-patterned film, the O*1s* peaks were convoluted to 529.8 and 531.9 eV. Following the wet-etching process, the intensity ratio of the Zn-O-related *O1s* peak to the Zn-*V*_*O*_-related *O1s* peak decreased, due to the increased surface area and porosity. The same ratio increased for both the NB and NR films following the additional growth step, implying that the ratio of crystal defects in the ZnO crystal structure were reduced during hydrothermal growth. It is also supported from Figure 4c, which shows that the binding energy of *Zn2p3* was shifted from Zn^2+^ to ZnO binding energy [26]. The higher value of the ratio for the NB film than the NR film can be attributed to the lower surface area of NB film.

**Figure 5:**
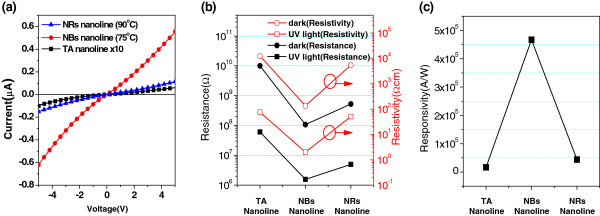
**Electrical properties of single TA, NB, and NR nanolines. (a)** Current-voltage (I-V) characteristic of single TA, NB, and NR ZnO nanolines in a dark environment, **(b)** change in their electrical resistance and electrical resistivity, and **(c)** their responsivity to illumination by UV light.

We characterized the electrical properties of single TA, NB, and NR nanolines fabricated on SiO_2_/Si substrates, and the results are shown in Figure 5. Figure 5a shows current-voltage (*I-V)* curves of all three single ZnO nanolines measured in a dark environment. Electrical resistance was smaller in the NB and NR nanolines than in the TA nanoline. Electrical resistance (*R*) can be formulated as *R* = *ρL*/*A* where *ρ* is electrical resistivity, *L* is the length of a sample, and *A* is the cross-sectional area normal to the direction of electron flow. From the equation, the reduced resistance could be attributed to both the enlarged cross-sectional area caused by additional growth (Figure 2) and the decreased resistivity. The electrical resistivity, *ρ,* was calculated using the above equation and is depicted in Figure 5b. The *ρ* of the NB and NR nanolines was reduced to 136 and 5,380 Ωcm, which is 1/90.1 and 1/2.3 of the TA nanoline, respectively. This result can be attributed to the increase in carrier concentration due to the filling of voids in the TA nanolines with nanocrystalline ZnO during the additional growth step, indicating that this process was key to improving electrical resistivity.

The photoresponse of various single-ZnO nanolines was also measured with illumination by UV light and is depicted in Figure 5b,c. Electrical resistance and resistivity both decreased upon illumination with UV light. The mechanism of photodetection is well known [6]. Oxygen molecules are adsorbed onto the surface of ZnO while capturing free electrons, forming a low-conductivity depletion layer near the surface. When illuminated by UV light, oxygen molecules are desorbed; electron-hole pairs are generated inside of ZnO; and the holes migrate to the surface and are trapped, leaving behind unpaired electrons to contribute to the photocurrent. When UV light is not present, oxygen molecules are re-adsorbed on the surface, retrapping free electrons, and the resistance is recovered.

Responsivity, defined as the ratio of the change of electrical current to the illuminated UV power, was calculated to be 1.66 × 10^4^, 4.67 × 10^5^, and 4.41 × 10^4^(A/W) for the single TA nanoline, NB nanoline, and NR nanoline, respectively. The responsivity of the NB nanoline was superior to the TA and NR nanolines. This result is noteworthy because both the electrical conductivity and responsivity of the ZnO nanoline were enhanced in the NB nanoline by the interior-architecturing, without the use of a high-temperature process of over 500°C [27]. We believe that fewer voids and interfacical trap states in the NBs nanoline led to an increase in the number of electron-hole pairs generated during UV illumination, to a greater extent than in the TA nanoline. In the case of the NR nanoline, its responsivity was almost as low as the TA nanoline. This result can be attributed to increased carrier scattering by the complex path in NR nanolines as well as increased grain boundaries due to augmented junctions between nanorods [28].

**Figure 6:**
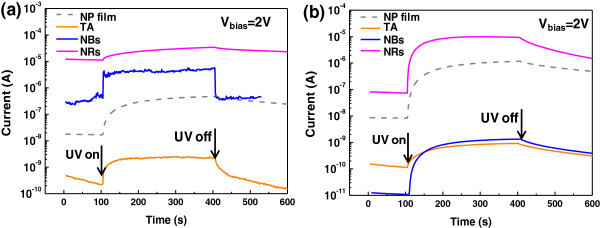
**Time-current responses to illumination of the devices by UV light.** Time-current response of the ordered arrays of overall 2,000 parallel TA, NB, and NR nanolines under illumination by UV light, fabricated on different substrates of **(a)** SiO_2_/Si and **(b)** PI.

We adopted the array of the ordered parallel ZnO nanolines to fabricate nanodevices with two electrodes by using simple deposition of Ag electrodes. Adopting the array of ZnO nanolines opens the possibilities of large-area, high-volume fabrication of ZnO nanostructure-integrated devices. Time-current responses to illumination of the devices by UV light at a bias voltage (*V*_bias_) of 2 V were recorded and are depicted in Figure 6. About 2,000 parallel ZnO nanolines were engaged to each nanodevice, and TA line-patterned ZnO film was tested together for comparison. Under illumination by UV light, the current through all the samples increased, agreeing with the result in the single nanolines. It is confirmed that arrays of ZnO nanolines can be effortlessly integrated with nanodevices, and function as UV photodetectors as effectively as a single ZnO nanoline. In the case of the SiO_2_/Si substrate (Figure 6a), the array of NB nanolines showed the shortest rising and falling time among the tested samples, due to easy adsorption and desorption of oxygen molecules as compared to the TA and NR nanolines. The NP film showed the longest falling time owing to the residual layer. Removal of the residual layer by the wet-etching process is thus seen to contribute largely to an improved response time. The current level was higher for the arrays of NB and NR nanolines due to the higher degree of crystallinity. In short, it is evident that the arrays of NB nanolines possess the optimum characteristics among the tested samples.

**Figure 7:**
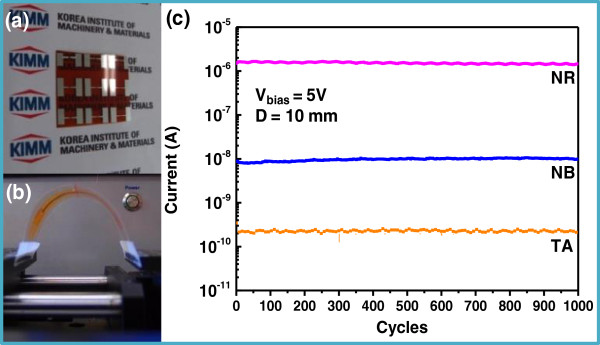
**Mechanical robustness of flexible devices with TA, NB, and NR nanolines respectively under 1,000 times cyclic loading.** Photo of **(a)** a flexible nanodevice and **(b)** bent flexible nanodevice during cyclic mechanical bending, and **(c)** electrical current measured during cyclic loading at 5-V bias voltage.

One of the great advantages of NIL is the ease of fabrication of nanostructures on flexible polymer substrates. The entire fabrication process was repeated on PI substrates, resulting in flexible ZnO nanostructure-integrated nanodevices. The electrical response of the array of ZnO nanolines on the PI substrate to UV light was measured and is shown in Figure 6b. It is noteworthy that the on-off ratio of the electrical current of flexible devices was ten times greater than rigid devices, owing to the lower current under a dark environment for flexible devices. This can be explained by two reasons which are a good dielectric quality of PI substrate [29] and an interfacial trap state in polymer (PI) and metal oxide (ZnO nanolines) [30] uneven thermal distribution and surface characteristics thus trapped electrons, and resultantly lowered current [31]. For those reasons, the falling time was deteriorated for the array of NB and TA nanolines compared with SiO_2_/Si substrate, but not in the case of NR nanoline, caused by its good electrical characteristics due to high crystalline as shown in Figure 3 and Figure S2 in Additional file 2. It means that readsorption of oxygen with UV light being turned off was easily facilitated for NR nanolines but hindered for NB and TA nanolines structure. SEM images in Figure S2 in Additional file 2 show smaller ZnO nanostructures on the PI substrate after hydrothermal synthesis as compared to the SiO_2_/Si substrate. The length of nanorods on the PI substrate was also shorter than on the SiO_2_/Si substrate, and could reduce access to and re-adsorption of oxygen compared with the SiO_2_/Si substrate.In order to verify the mechanical robustness of the ZnO nanoline-integrated flexible devices, a mechanical bending test was performed using 1,000 repetitions of cyclic bending, and the electrical current of the devices was measured during the experiment, as shown in Figure 7. All the tested devices showed negligible change in current and excellent robustness upon being bent 1,000 times for 10 mm in ambient air.

## Conclusions

In this paper, interior-architecturing of ZnO nanostructure was demonstrated to improve its electrical characteristic processed at low temperature by using stepwise fabrication process. Investigation of the structural morphology and crystallographic orientation confirmed that interior structure of ZnO nanostructure was changed as filling of voids with newly growing nanocrystalline ZnO during the fabrication process and the change contributed to the improvement in electrical characteristics. The fabricated ZnO nanolines also showed good electrical responses to illumination by UV light.

The arrays of ZnO nanoline were easily implemented on both rigid and flexible substrates and functioned as UV-sensitive devices with good sensitivity. Interior-architecturing of ZnO nanostructure lends itself well to tunability of morphological, electrical, and optical characteristics of nanopatterned inorganic materials with the large-area, low-cost, and low-temperature producibility. Furthermore, all experimental nano arrays on PI films showed outstanding robustness under prolonged bending tests and, in combination with printing technology, could be widely applied to next-generation flexible applications.

## Competing interests

The authors declare that they have no competing interests.

## Authors’ contributions

EC and JL carried out the data processing, image processing, and analysis and wrote the manuscript. SK, JHC, and DGC produced the resin and mold for testing and participated in the sample test. JYJ prepared the films and tested the surface topography by XRD and XPS. The optical properties and bending test were measured by JL and IP. Besides, JHJ helped to draft the manuscript and prepared nanoimprint facility. All authors read and approved the final manuscript.

## Supplementary Material

Additional file 1: Figure S1 **SEM images of Banana-bundle-like ZnO nanostructure.** SEM images of Banana-bundle-like ZnO nanostructure fabricated on the line-patterned ZnO film with residual layer by additional growth at 90°C. Click here for file

Additional file 2: Figure S2 **Result of hydrothermal synthesis on PI at 75°C ( a) and 90°C (b) for 30 mins.** The average width of NBs and NRs is about 50 nm and 70 nm, respectively, which is smaller than those of same structures on Si substrates processed under the same condition. The overall size of the nanostructures was smaller, and thus insufficient filling of voids inside the nanostructure and slow re-adsorption of oxygen can be presumed for NBs nanolines on the PI substrate. Additionally, the exceptionally low current level of the array of NBs nanolines on the PI substrate under UV illumination is also a result of insufficient filling and low connectivity among nanograins. Hence it appears that the array of NRs nanolines is better suited to integration on PI substrates for photodetection. Click here for file
