# Supplementary figures and images for: Interior-architectured ZnO nanostructure for enhanced electrical conductivity via stepwise fabrication process
Source: Nanoscale Res Lett. 2014 Aug 24;9(1):428. doi: 10.1186/1556-276X-9-428 (PMC4165434; doi:10.1186/1556-276X-9-428)

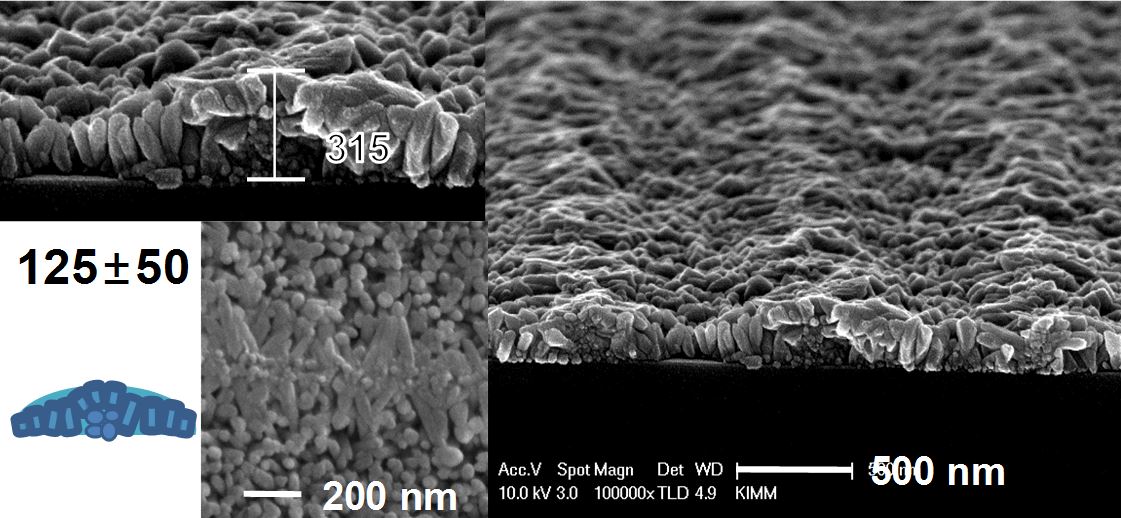

Supplement: Additional file 1: Figure S1 — SEM images of Banana-bundle-like ZnO nanostructure. SEM images of Banana-bundle-like ZnO nanostructure fabricated on the line-patterned ZnO film with residual layer by additional growth at 90°C. [file 1556-276X-9-428-S1.jpeg]

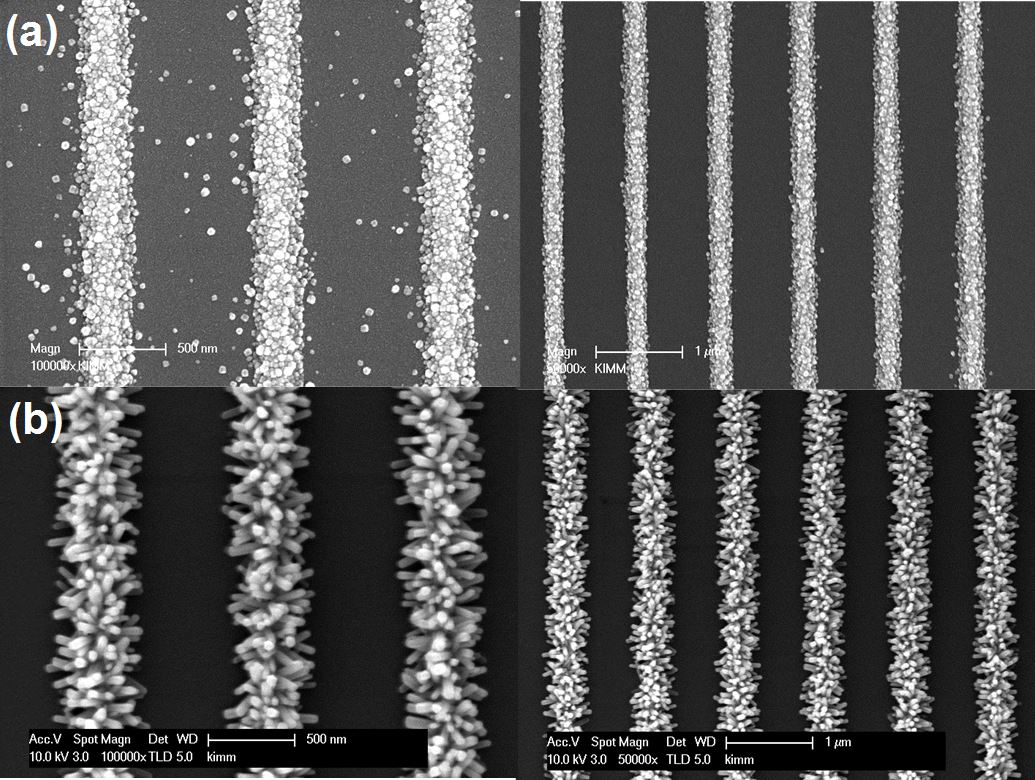

Supplement: Additional file 2: Figure S2 — Result of hydrothermal synthesis on PI at 75°C ( a) and 90°C (b) for 30 mins. The average width of NBs and NRs is about 50 nm and 70 nm, respectively, which is smaller than those of same structures on Si substrates processed under the same condition. The overall size of the nanostructures was smaller, and thus insufficient filling of voids inside the nanostructure and slow re-adsorption of oxygen can be presumed for NBs nanolines on the PI substrate. Additionally, the exceptionally low current level of the array of NBs nanolines on the PI substrate under UV illumination is also a result of insufficient filling and low connectivity among nanograins. Hence it appears that the array of NRs nanolines is better suited to integration on PI substrates for photodetection. [file 1556-276X-9-428-S2.jpeg]
